# Metabolic Barriers to Glioblastoma Immunotherapy

**DOI:** 10.3390/cancers15051519

**Published:** 2023-02-28

**Authors:** Nikita Choudhary, Robert C. Osorio, Jun Y. Oh, Manish K. Aghi

**Affiliations:** Department of Neurosurgery, University of California San Francisco, San Francisco, CA 94143, USA

**Keywords:** glioblastoma, immunotherapy, metabolism, tumor microenvironment, glycolysis, glutamine metabolism, lipid metabolism, tryptophan metabolism

## Abstract

**Simple Summary:**

Glioblastoma (GBM) is an aggressive brain tumor with limited prognosis despite multimodal treatment approaches. Various immunotherapies have been investigated to address the need for novel therapeutic options in GBM with limited success. Recently, alterations in the metabolism of cancer cells which allow for tumor proliferation, but simultaneously alter immune populations leading to an immunosuppressive tumor microenvironment, have been investigated as contributory to therapeutic resistance. This review discusses metabolic alterations in GBM tumor cells which have been investigated as contributory to immunosuppression and resistance to immunotherapies.

**Abstract:**

Glioblastoma (GBM) is the most common primary brain tumor with a poor prognosis with the current standard of care treatment. To address the need for novel therapeutic options in GBM, immunotherapies which target cancer cells through stimulating an anti-tumoral immune response have been investigated in GBM. However, immunotherapies in GBM have not met with anywhere near the level of success they have encountered in other cancers. The immunosuppressive tumor microenvironment in GBM is thought to contribute significantly to resistance to immunotherapy. Metabolic alterations employed by cancer cells to promote their own growth and proliferation have been shown to impact the distribution and function of immune cells in the tumor microenvironment. More recently, the diminished function of anti-tumoral effector immune cells and promotion of immunosuppressive populations resulting from metabolic alterations have been investigated as contributory to therapeutic resistance. The GBM tumor cell metabolism of four nutrients (glucose, glutamine, tryptophan, and lipids) has recently been described as contributory to an immunosuppressive tumor microenvironment and immunotherapy resistance. Understanding metabolic mechanisms of resistance to immunotherapy in GBM can provide insight into future directions targeting the anti-tumor immune response in combination with tumor metabolism.

## 1. Introduction

Glioblastoma (GBM) is the most common primary brain tumor with a limited prognosis and a median survival of 15 months despite an aggressive standard of care treatment consisting of maximal safe surgical resection followed by radiation and chemotherapy with temozolomide [1]. Since the addition of temozolomide to the standard of care treatment in 2005, subsequent efforts to develop new therapeutic candidates have failed to outperform standard of care treatment in clinical trials [2]. Developing effective novel therapies for GBM therefore remains an unmet need.

One novel emerging area of cancer therapeutics is immunotherapies, which target one of the hallmarks of cancer—the ability to evade cellular immunity that would otherwise result in immunological targeting of tumor cells [3]. While in recent years immunotherapies have become standard treatment options for several cancer types, a variety of immune-based therapies including checkpoint inhibitors, vaccines, CAR-T cells, oncolytic viruses, and myeloid-targeted therapies have failed to benefit patients with GBM in trials [4]. The uniquely immunosuppressive tumor microenvironment in GBM is thought to contribute significantly to immunotherapy resistance [4,5]. 

The tumor microenvironment is affected by unique cancer cell metabolism that not only promotes tumor cell growth but also alters the pH, oxygen, and metabolite contents that affect the survival and function of immune cells in the tumor microenvironment [6]. Metabolic reprogramming within tumor cells diminishes the function of effector immune cells through depletion of essential metabolites and promotes enrichment of suppressive immune populations [7]. More recently, therapies targeting metabolic factors in the tumor microenvironment that adversely impact the antitumor immune response such as low glucose, low pH, hypoxia, and the generation of suppressive metabolites have been explored as immunotherapeutic anticancer strategies [7]. Similar findings have also been reported in GBM, where metabolic reprogramming in tumor cells plays a significant role in driving survival, proliferation, and invasion. However these metabolic adaptations additionally alter the GBM tumor immune microenvironment [8]. In this review, we discuss how GBM tumor cell metabolism of four nutrients (glucose, glutamine, tryptophan, and lipids) leads to an immunosuppressive tumor microenvironment and the implications of these metabolic changes on immune based treatment strategies for GBM. 

## 2. Effects of Increased Tumor Cell Reliance on Glycolysis on the Immune Microenvironment of Glioblastoma

Glycolysis is the most prominent metabolic pathway implicated in cancer metabolism as contributory to sustaining the energetic cost of growth and proliferation. During glycolysis, glucose is catabolized to pyruvate which is then converted to lactate to either be secreted or enter the TCA cycle generating ATP and NADH in the process. Glucose metabolism plays a significant role in the brain microenvironment given the high metabolic demand of the brain and lack of glycogen storage within the brain. High blood glucose levels and increased neuronal expression of glucose transporters have been linked to decreased survival in glioblastoma patients [9]. 

Altered glucose metabolism in tumor cells results in preferential aerobic glycolysis—increased glycolytic activity despite the presence of oxygen enabling alternate metabolic pathways, a phenomenon known as the Warburg effect. Though glycolysis is an inefficient pathway for energy production relative to mitochondrial oxidation, increased glycolysis in proliferating tumor cells generates metabolic precursors such as lactate which are thought to be the rate-limiting factors during cellular proliferation. In tumor cells, glucose transporters and glycolytic enzymes essential for the conversion of pyruvate to lactate are upregulated. In glioblastoma, the significantly increased rate of glycolysis drives energy production [10]. Tumor cells develop alterations to allow for this increased glycolysis and tumor growth [10]. Genome-wide transcriptomic analysis of patient-derived GBM cells demonstrate strong upregulation of glycolysis-related genes [11]. Hexokinase 2 (HK2), an isoform of the enzyme which catalyzes the conversion of glucose to glucose-6-phosphate in the first step of glycolysis is strongly expressed in GBM [12]. Knockdown or silencing of glycolytic genes such as *HK2*, *PKFP*, *ALDOA*, *PGAM1*, *ENO1*, *ENO2*, or *PDK1* inhibits GBM tumor growth and prolongs survival in a mouse xenograft model [11]. These differentially regulated genes were involved in glycolysis and downstream hypoxia response signaling pathways, suggesting that the glycolytic enzymes encoded by these genes are essential for GBM growth [11]. 

**Figure 1:**
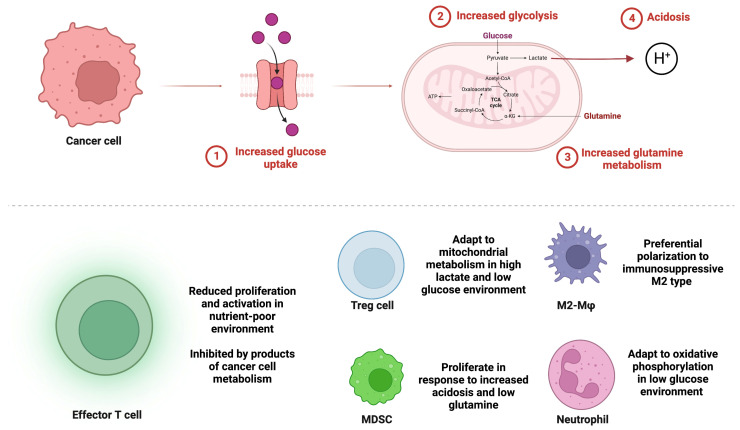
Glucose and glutamine metabolism in cancer cells alters immune cell populations in the glioblastoma tumor microenvironment. (**Top**) Cancer cells up regulate glucose transport into the cell (**1**) and increase glycolytic (**2**) and glutamine metabolism (**3**) leading to low levels of glucose and glutamine in the tumor microenvironment. Increased glycolysis results in acidosis from increased lactic acid production (**4**). (**Bottom**) Immune cell populations respond differentially to metabolic alterations in the tumor microenvironment. Anti-tumoral effector T cells have reduced function and proliferation. Pro-tumoral immunosuppressive populations of Tregs, MDSCs, M2 polarized macrophages, and neutrophils are enriched.

More recently, attention has been given to the impact of alterations in glycolytic pathways on not only proliferating tumor cells but also the tumor microenvironment and resulting changes in immune cell metabolism and function (Figure 1) [13]. Glycolysis alters the immune response in cancer as shown by glycolysis-related genes with prognostic value found to be linked to varying immune cell infiltration and differential immune-related gene expression [14].

Glycolysis requires export of lactate from cells by transporters which co-transport lactate and protons (H+), leading to their accumulation in the tumor microenvironment and resulting in tumor acidosis which impacts the function of immune cells in the tumor microenvironment. Acidosis has been described in other cancers as contributory to immunosuppression [15]. Lactic acid produced by tumor cells inhibits the differentiation and activation of monocytes and T cells and regulates the expression and secretion of tumor-promoting cytokine interleukin 23 [16,17]. Lactate accumulation additionally inhibits type 1 interferon signaling and granzyme B expression which normally promotes cancer immunosurveillance through the activity of natural killer (NK) cells [18,19]. In melanoma, lactic acid production by tumor cells reduces the quantity and the cytotoxic activity of CD8 T cells and NK cells in culture and in vivo [20]. Activated T cells require the ability to co-transport lactate and protons as part of their own glycolytic metabolism. Increased lactic acid production by tumor cells has been shown to inhibit T cell glycolysis and function by altering the concentration gradient for lactate and proton export by the T cells [21]. In effect, increased glycolysis in tumor cells inhibits the ability of T cells to engage in glycolytic metabolism [20]. 

Strategies that free T cell glycolytic metabolism from the restrictions imposed on these cells by the tumor microenvironment have been evaluated in preclinical models. For example, genetic modification of tumor specific CD4 and CD8 T cells to overexpress phosphoenolpyruvate carboxykinase 1 (PCK1) increased the production of the glycolytic metabolite phosphoenolpyruvate, resulted in increased T cell glycolysis, increased T cell effector function, and restricted tumor growth and prolonged survival in a melanoma mouse model [22]. Further supporting the idea that increased tumor glycolysis results in a glucose-poor tumor microenvironment that diminishes T cell function, increased glycolytic metabolism in melanoma cells has been associated with resistance to adoptive T cell therapy and checkpoint blockade [23]. Another study in a mouse sarcoma model demonstrated that glucose consumption by tumors leads to metabolic restriction of T cells and reduced T cell glycolytic capacity allowing for tumor progression [24]. In this study, an antigenic model that enhanced glycolysis of T cells led to slower tumor growth [24]. Calcinotto et al. demonstrated that increased acidosis resulted in mouse and human CD8 T cell anergy [25]. Combining proton pump inhibitors lowering pH with adoptive transfer of antigen specific T cells or vaccines to melanoma specific antigens resulted in increased therapeutic efficacy in a mouse model of melanoma [25]. In a separate study of mouse models of multiple cancer types, neutralizing tumor acidity increased T cell infiltration and impaired tumor growth [26]. Furthermore, combining bicarbonate therapy for neutralization of tumor acidosis with checkpoint inhibitors or adoptive T cell transfer improved antitumor responses [26].

Lactic acid production has also been suggested to not only reduce anti-tumoral immune cell populations but also promote immunosuppressive populations. Notably, myeloid cells are resistant to lactic acid-induced apoptosis [20]. In fact, in some studies, these cells have not only been resistant to the effects of lactic acid, but the most aggressive pro-tumoral myeloid cells often thrive in response to lactic acid. For example, accumulation of lactic acid in pancreatic tumor cells was shown to increase the number of myeloid derived suppressor cells (MDSCs) in mice [27]. Colegio et al. showed that lactic acid promotes polarization of macrophages towards the tolerogenic M2 type [28]. Suppressive Treg cells are not impaired by the low lactate levels that impair the function of effector T cells. In fact, Treg cells are able to generate NAD+ through mitochondrial metabolism in high lactate environments [29].

Glycolytic alterations may also specifically impact neutrophils. While less is understood about the metabolic utilization of neutrophils in the tumor microenvironment than leukocytes, neutrophils are generally regarded as highly glycolytic. Neutrophil function has been described to highly depend on glucose availability with lack of glucose abrogating function [30]. suggesting that increased glucose metabolism by tumor cells may limit the availability of glucose to neutrophils limiting their function. However, counterintuitively to a perhaps reduced function in a tumor microenvironment with low levels of glucose, neutrophil recruitment to the tumor site has been regularly described as immunosuppressive and inhibitory of the activity of T cells [31]. An elevated circulating neutrophil to lymphocyte ratio (NLR) has been found to be a negative prognostic factor in glioblastoma patients [32]. Rice et al. suggest a potential mechanism in which neutrophils maintain local immune suppression in the glucose-limited tumor microenvironment through the adaptation of a neutrophil subpopulation to an oxidative mitochondrial metabolism [33].

Interestingly, the interplay between glycolytic tumor metabolism and immune cell function may be bidirectional with immune cells able to regulate metabolic pathways as well. Zhang et al. show that macrophages produce interleukin-6 which leads to downstream phosphorylation of the glycolytic enzyme phosphoglycerate kinase 1 (PGK1) and facilitates a PGK1-catalyzed reaction towards glycolysis rather than gluconeogenesis through altered substate affinity [34]. PGK1 phosphorylation correlated with increased macrophage infiltration, higher grade, and worse prognosis in human GBM samples [34]. Further work will be necessary to elucidate this metabolic crosstalk and metabolic competition between tumor cells and immune cells and to understand whether immune cells can themselves alter the metabolic environment to support tumor growth, including through mechanisms, such as post-translational modifications, which regulate the functions of many glycolytic enzymes. 

Studies of glycolysis in glioblastoma have paralleled the findings in other cancer types of the significance of increased glycolysis in creating an immunosuppressive tumor microenvironment. The shift to increased aerobic glycolysis from oxidative phosphorylation in glioblastoma is associated with immunosuppression and tumor progression [35]. In GBM, hypoxia-inducible factor 1α (HIF-1α) directs the metabolic switch for Tregs from glycolysis in the glucose-poor tumor environment to oxidative phosphorylation which drives immunosuppression [36]. One recent study determined a glycolytic score for glioblastoma utilizing seven genes involved in expression of glycolytic enzymes and found that T cells, B cells, and NK cells were depressed while there was high infiltration of immunosuppressive cells in patients with high glycolytic scores [37]. One of the genes utilized in the glycolytic score, *ENO1*, promoted M2 microglia polarization promoting immunosuppression and glioblastoma cell malignancy. Another recent study utilizing differentially expressed genes between high and low glycolytic activity to assign risk scores to classify high and low risk GBM patients found differential infiltration of immune cells and immune checkpoints, suggesting a relationship between glycolytic activity and immunosuppression in patients with GBM [38]. 

## 3. Anti-Tumoral Immunologic Effects of Targeting Glutamine Metabolism in Cancer

Glutamine, an amino acid highly expressed in cancer cells, plays a critical role for cellular function and the generation of energy and metabolic precursors for macromolecule synthesis which help sustain anabolic growth. Glutamine is converted by glutaminase into glutamate which is then converted to α-ketoglutarate, a critical component of the TCA cycle and in the production of metabolic intermediates utilized in the production of lipids, nucleic acids, and proteins. Upregulated glutamine metabolism in cancer cells promotes tumor growth through supporting macromolecule biosynthesis, altered signaling pathways, and cancer cell proliferation and survival. The metabolism of glutamine provides carbons for the TCA cycle to sustain accelerated anabolism in cancer cells and promotes tumor growth [39,40,41].

Glutamine is amongst the most prevalent amino acids in the brain as a precursor to the excitatory neurotransmitter glutamate [9]. Glutamate transporters are upregulated in gliomas allowing for increased glutamine uptake [42]. The absence of glutamine in culture medium leads to loss of viability as determined by a trypan blue dye exclusion test in glioma cell lines [43]. Increased and rapid glutamine utilization has been described as characteristic of glioblastoma cell proliferation through promoting generation of NADPH for anabolic processes such as nucleotide biosynthesis and providing a source of carbon for fatty acid synthesis [44]. Glutamate secretion in glioma cells results in a growth advantage in vivo, and targeting glutamate secretion or antagonizing glutamate target receptors resulted in slowed tumor expansion in C6Glu+ tumors in rats [45]. 

Glutamine metabolic pathways are also upregulated in glioblastoma. Glutamate dehydrogenase (GDH), an enzyme which catalyzes the conversion of L-glutamate into α-ketoglutarate as part of glutaminolysis, is upregulated in many human cancers and shown to promote tumor growth [46]. An isoenzyme of GDH, GDH1, maintains glioma cell survival in glucose depleted conditions through activation of glutamine metabolism and the α-ketoglutarate generated drives glucose uptake and cell survival under low glucose [47]. Glutamine synthetase expression in glioblastoma is associated with poor prognosis, with absent or low intensity expression of glutamine synthetase in neoplastic cells associated with longer survival [48]. Glutamine is hypothesized to be provided by surrounding astrocytes to feed GBM cells negative for glutamine synthetase cells [49].

Glutamine metabolism in cancer cells impacts the tumor microenvironment and the immune populations within it in ways similar to glucose metabolism (Figure 1). Cancer cells relying on exogenous glutamine synthesis utilize glucose, further depleting it in the tumor microenvironment and contributing to the reduced function of immunostimulatory effector T cells and NK populations that require glucose for function [7]. Glutaminolysis also results in the downstream production of lactate, mirroring the effect of aerobic glycolysis in generating an acidic tumor microenvironment which contributes to immunosuppression as described earlier in this review. 

Increased uptake of glutamine by tumor cells may result in its depletion in the tumor microenvironment and affect the function of immune cells which utilize glutamine for their own metabolic programs. Activated T cells upregulate glutamine metabolism to generate α-ketoglutarate to enter the TCA cycle and generate ATP to fulfill the energetic demands of T cell proliferation [50]. Glutamine depletion in the tumor microenvironment compromises activation-induced T cell growth and proliferation. Addition of the macromolecular products of glutamine synthesis (nucleotides and polyamines) does not rescue T cell growth in a glutamine depleted environment, implicating the specific role of glutamine in meeting the bioenergetic and biosynthetic precursor requirements of activated T cells [50]. 

Targeting glutamine metabolism in a mouse model of colon cancer through a glutamine antagonist 6-Diazo-5-oxo-L-norleucine, which broadly inhibits several glutamine-using enzymes, led to suppression of both oxidative phosphorylation and glycolytic metabolism in cancer cells and decreased tumor-related changes in the microenvironment with decreased hypoxia, acidosis, and nutrient depletion [51]. In contrast, glutamine blockade in effector T cells resulted in upregulated oxidative metabolism and increased survival and activation [51]. PD-1-targeted checkpoint blockade co-administered with glutamine antagonism resulted in a complete therapeutic response and a memory response with tumor rechallenge [51]. The efficacy of glutamine antagonism was entirely dependent on the activity of CD8 T cells, indicating that the mechanism through which glutamine antagonism promoted anti-tumoral activity was through enhancing cytotoxic T cell anti-tumor response [51]. These findings highlight a common theme for glutamine and glycolytic metabolism in which tumor cells and anti-tumor immune cells compete for metabolites to promote their individual function. It additionally offers insights for metabolic targeting in cancer that leverages the therapeutic window created by the differential metabolic plasticity of immune cells versus cancer cells in which cancer cells are highly metabolically interdependent (targeting glutamine metabolism leads to widespread metabolic inhibition), whereas T cells exhibit adaptive metabolic reprogramming (targeting glutamine metabolism activates upregulation of alternate pathways allowing survival). 

Additionally, glutamine metabolism by cancer cells leads to the enrichment of various immunosuppressive populations in cancer. Notably, α-ketoglutarate generated through glutaminolysis restricts anti-tumoral macrophage M1 activation [52]. A separate study also supports the role of α-ketoglutarate in promoting an immunosuppressive macrophage phenotype by showing that higher production of α-ketoglutarate results in M2 activation of macrophages (an immunotolerant phenotype) [53].

Targeting glutamine metabolism in cancers with known resistance to checkpoint blockade (triple negative breast cancer and lung carcinoma) with a small molecule inhibitor led to the marked inhibition of the generation and recruitment of immunosuppressive myeloid-derived suppressor cells (MDSCs) through apoptosis of these MDSCs [54]. Additionally, glutamine antagonism promoted the generation of antitumor inflammatory tumor-associated macrophages [54]. Combining glutamine antagonism with checkpoint blockade in immunotherapy-resistant tumors was shown to enhance the efficacy of checkpoint blockade [54].

Targeting glutamine metabolism may enhance endogenous anti-tumor immunity through independent mechanisms promoting the metabolic programs of cytotoxic populations while inhibiting immunosuppressive populations. Given the success of combining targeting glutamine metabolism with checkpoint inhibitors in other immunotherapy resistant tumors, it may be worthwhile to explore this combination in GBM. 

## 4. Inhibition of Tryptophan Degrading Enzymes as a Strategy to Promote an Anti-Tumoral Immune Response

Tryptophan is an essential amino acid utilized for protein biosynthesis and a biochemical precursor to physiologically important compounds such as serotonin and melatonin. The majority of tryptophan which is not incorporated into proteins is broken down into degradation products (kynurenines) via the kynurenine pathway [55]. Physiologically, tryptophan degradation into kynurenines enables the generation of the essential metabolic cofactor nicotinamide adenine dinucleotide (NAD+). In cancer, the production of kynurenine metabolites by tumor cells contributes to tumor growth by generating an immunosuppressive tumor microenvironment through the recruitment and differentiation of immunosuppressive Treg cells and MDSCs [56]. Tryptophan is degraded into kynurenine metabolites by the two enzymes indoleamine-2,3-dioxygenase-1 (IDO1) or tryptophan-2,3-dioxygenase (TDO2) which catalyze the rate limiting reactions in the kynurenine pathway. 

Tryptophan catabolism and alterations in kynurenine pathway has been implicated in poor prognosis in several cancer types, including in GBM [57]. The correlation between overexpression of enzymes involved in tryptophan degradation and patient survival in primary and metastatic brain tumors has been well established [58,59]. Recurrent malignant gliomas are associated with increased levels of tryptophan metabolism compared to newly diagnosed patients in metabolic profiles obtained from CSF analysis [60]. IDO1 and TDO2 are highly expressed in glioma cells proportionally to glioma grade [55,61]. Additionally, amongst higher grade patients, those with strong IDO expression were noted to have significantly worse overall survival rates compared to patients with weak IDO expression [61]. IDO1 is expressed in the majority of malignant gliomas with mRNA and protein expression levels correlating with overall patient survival [59].

**Figure 2:**
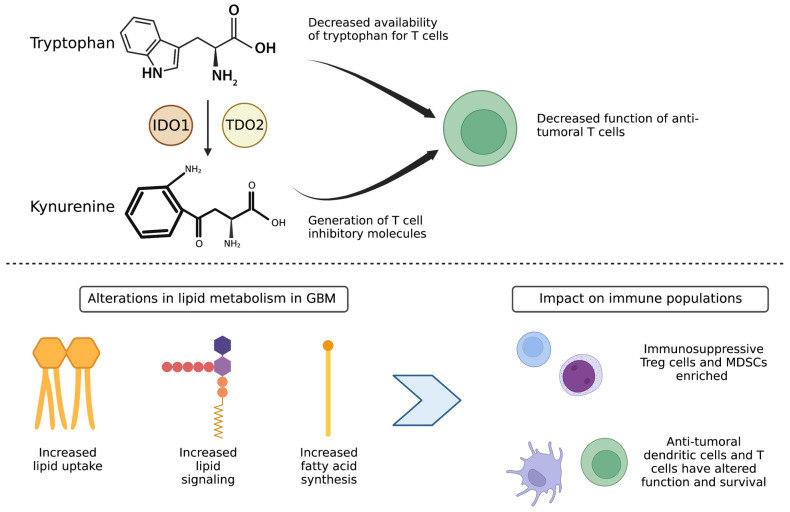
Altered tryptophan metabolism and lipid metabolism in GBM tumor cells contributes to an immunosuppressive population enriched tumor microenvironment. (**Top**) Tryptophan metabolism and generation of kynurenine degradation products by the enzymes IDO1 and TDO2 leads to decreased availability of tryptophan for T cells and generation of T cell inhibitory molecules, leading to decreased function of anti-tumoral T cells. (**Bottom**) Alterations in lipid metabolism lead to increased fatty acid oxidation and lipid signaling by immunosuppressive Treg and MDSC cells, leading to their enrichment over anti-tumor dendritic cells and cytotoxic T cells.

IDO1- and TDO2- mediated degradation of tryptophan by cancer cells is a driver of immune suppression in the tumor microenvironment through recruitment and activation of myeloid-derived suppressor cells (MDSCs) and induction of anergy of CD8+ T cells [62]. Degradation of tryptophan and reduced tryptophan availability within the tumor microenvironment resulting in arrest of T cell growth and activation has been well characterized [63]. Tryptophan-free media suppresses human T cell proliferation and activation [64]. Tryptophan catabolism by tumor cells allows for metabolic inhibition of T cells and promotes tumor evasion of immune destruction. Two mechanisms enable tumor cell tryptophan catabolism to inhibit anti-tumoral T cells: (1) tumor cell depletion of the essential metabolite tryptophan which is required for T cell metabolism (the competition for nutrients scenario between tumor cells and T cells described above for glucose and glutamine) and (2) generation of T cell inhibitory molecules from tryptophan metabolites such as kynurenine and its derivatives (Figure 2).

Tryptophan utilization by tumor cells leads to metabolic starvation of T cells which are unable to utilize tryptophan for their own functions and thus promotes immunosuppression in the tumor microenvironment. Inhibition of tryptophan degrading enzymes blocks enzymatic activity and restores cytotoxic T cell activity in vitro and in vivo [65]. T cells undergo rapid growth arrest in low tryptophan conditions due to a tryptophan-sensitive checkpoint inhibiting the cell cycle in the G1 phase [66]. High IDO expression in colorectal cancer cell lines was associated with significant reduction of CD3+ infiltrating T cells and increased frequency of liver metastases [67]. Additionally, intratumoral immunosuppressive cells—such as MDSCs, tumor associated macrophages (TAMs), and Treg cells—upregulate production of IDO and metabolize tryptophan into suppressive kynurenine which reduces the availability of tryptophan for cytotoxic T cells in the tumor microenvironment [68]. This mechanism is so crucial to MDSC immunosuppression that IDO has been shown to be required for MDSCs’ immunosuppression of T cells with inhibition of IDO leading to decreased MDSC suppression of T cell proliferation in a murine melanoma model [68,69].

Given the role of IDO through kynurenine synthesis in generating the tumor microenvironment allowing for immune escape in cancer, IDO inhibition has been explored as an attractive therapeutic option in multiple cancers. Inhibition of IDO was found to effectively normalize plasma kynurenine levels in patients with various tumor types [70]. Interestingly, combinatorial inhibition of IDO1, IDO2, and TDO2 (together thought to be the predominant rate-limiting enzymes for the kynurenine pathway) did not impact tumor viability in patient derived GBM cells [55]. However, these findings are consistent with the mechanistic understanding that inhibition of the kynurenine pathway enzymes has anti-tumoral effects due to alterations in the survival and function of immune cells normally present in the tumor microenvironment that are not present in an in vitro model. 

Kynurenine metabolites activate a ligand-activated transcription factor, aryl hydrocarbon receptor (AhR) which results in increased expression of IDO1 and IDO2 in a positive feedback loop. Targeting AhR in vitro led to decreased glioma cell viability [55]. Opitz et al. also showed that kynurenines generated by TDO act to suppress antitumor responses by T cell suppression and promotion of tumor cell survival through AhR mediated signaling in a murine glioma model [71]. Increased expression of AhR target genes involved in signaling pathways related to immune tolerance correlated with decreased survival in patients with glioma [71]. Additionally, AhR activity was found to drive T cell dysfunction through promotion of a Treg-macrophage suppressive axis [62]. This alternate pathway of AhR agonism may circumvent the anti-tumoral effects of tryptophan degradation inhibition through AhR agonism independent of immune function. This also suggests a potential limitation of previous clinical trials of IDO1 inhibitors, some of which have been AhR agonists themselves [72].

Tryptophan catabolism and its downstream metabolic pathways are known to contribute to the immunosuppressive tumor microenvironment and contribute to resistance to novel immunotherapies for malignant gliomas. Increased expression of IDO and TDO has been suggested as an acquired resistance mechanism to PD-1 and CTLA blockade in pre-clinical models of multiple cancers, including GBM [73,74]. The effect of CTLA-4 blockade synergized with IDO inhibitors in metastatic melanoma in both IDO-expressing and non-IDO-expressing poorly immunogenic tumors, and was shown to be effector T cell dependent [75]. 

The combination of targeting tryptophan metabolism with immune checkpoint blockade has been also explored in glioblastoma. Combining 1-methyltryptophan, which inhibits IDO, with dual immune checkpoint blockade significantly improved survival in an orthotropic mouse GBM model correlating with increased T-cell survival and synergistic decrease of Treg infiltration [76]. Likewise, immunotherapy simultaneously targeting IDO, CTLA-4, and PD-L1 in a mouse glioma model demonstrated a survival benefit [57]. However, combinatorial effects of IDO inhibition with checkpoint inhibitors have been observed in mouse models of other cancers but have not necessarily translated to success in clinical trials. Most prominently, a large phase 3 trial of an IDO1 enzyme inhibitor plus a PD-1 inhibitor in metastatic melanoma did not result in greater clinical benefit compared to PD-1 inhibition alone [77]. Subsequently, multiple phase 3 trials of various IDO1 inhibitors in combination with immune checkpoint inhibitors in other cancers were halted. 

Potential limitations of IDO1 inhibition that led to failure in this phase 3 trial combining this approach with a PD-1 inhibitor include: insufficient inhibition of IDO1 at the doses being used; the ability of other enzymes involved in tryptophan metabolisms such as TDO2 or pathways downstream of IDO1 inhibition to compensate and still generate immunosuppressive tryptophan metabolites such as kynurenine and its derivatives when IDO1 is inhibited; and lack of patient selection based on IDO1 expression [65,78]. IDO1 may also suppress the antitumor immune response independent of its association with tryptophan metabolism [79]. Additionally, while overwhelming evidence suggests that IDO expression and tryptophan degradation results in immunosuppression and T cell dysfunction diminishing the efficacy of immunotherapy, the understanding of IDO interaction with immunotherapy remains incomplete. Counterintuitively, brain-tumor mice genetically deficient for IDO1 demonstrate decreased efficacy in dual and triple immunotherapy approaches [57]. Thus, IDO inhibition or deficiency may be evaded by alternate metabolic pathways that maintain continued immunosuppression. One study demonstrated that while IDO1 was identified as the top gene in determining low versus high tryptophan in GBM, another potential mediator of the high tryptophan metabolic phenotype in GBM, quinolinate phosphoribosyltransferase, was identified as well, suggesting alternate pathways that could be upregulated to evade IDO1 inhibition and maintain the high utilization of tryptophan in tumor cells [80]. Interestingly, targeting AhR in tumors with an active tryptophan catabolic pathway allows for the overcoming of immunosuppression and sensitization to anti-PD-1 therapy, suggesting a role for targeting alternate areas of tryptophan metabolism [62]. More work is needed to understand the role of compensatory pathways in targeting tryptophan metabolism.

Targeting tryptophan metabolism may also have implications for vaccine related cancer therapies which rely on T cell mediated antitumoral responses. IDO expression correlated with lack of specific T cell enrichment at the tumor site and prevented the rejection of tumor cells in mice who have been preimmunized against tumor antigens with a vaccine [58]. This effect was partially reversible with systemic administration of an IDO inhibitor, holding implications for combining cancer vaccines which utilize tumor specific antigenic peptides to generate a T cell antitumor response with IDO inhibitors to enhance the antitumoral effect of immunomodulatory vaccines. 

## 5. Exogenous Induction of Lipid Peroxidation as a Strategy to Promote an Anti-Tumoral Immune Response 

Lipid metabolism physiologically functions to allow for cellular energy storage, synthesis of cellular membranes, and cellular signaling. In cancer, alterations in lipid metabolism help meet high bioenergetic demands by generating energy through beta-oxidation. Utilization of fatty acid oxidation in addition to increased glycolysis allows for bioenergetic flexibility in promoting aggressive tumor growth and metastasis [81]. 

Glioma cells utilize lipid oxidation and upregulate transport of ketones generated from lipid metabolism to sustain growth [82]. Lipid metabolism is abnormally regulated in gliomas with altered expression of lipid-related genes, altered lipid composition, and lipogenesis [83]. Lipids provide energy to fuel GBM cellular proliferation and also play a role in mitigation of oxidative damage that is increased during proliferation [84]. Evidence supporting the role of lipid metabolism in GBM biology comes from studies in which targeting lipid homeostasis inhibits GBM cell proliferation [85]. Lipids are also utilized through lipolysis to maintain stem cell self-renewal in GBM and to allow for propagation of orthotopic tumor xenografts from GBM stem cells in mice [86]. Differential expression of nine genes related to lipid metabolism has been shown to allow the classification of GBM patients into high and low risk for poor outcome [87].

Altered lipid metabolism in GBM impacts immune cell function, particularly that of T cells [88]. T cells utilize lipids to promote their proliferation and differentiation by taking up exogenous lipids and oxidizing intracellular stores of lipids [89]. In GBM, T cells are sequestered in the bone marrow away from the tumor microenvironment via T cell internalization of the lipid sphingosine-1-phosphate receptor, which has been suggested to play a protumoral role through promotion of angiogenesis in GBM [90]. Notably, Treg cells also primarily utilize fatty acid oxidation for their metabolism [91]. 

While less is understood about the metabolic requirements of Treg cells, utilizing fatty acid oxidation over glycolysis may promote Treg survival over the survival of CD4 and CD8 T cells (Figure 2). Lipid signaling in intratumoral Treg cells additionally allows for cell survival and induces signaling pathways to promote oxidative phosphorylation in Treg cells [92]. Another class of suppressive immune cells, MDSCs, have also been found to demonstrate increased fatty acid uptake and activated fatty acid oxidation in multiple murine tumor models [93]. Pharmacological inhibition of fatty acid oxidation led to decreased production of inhibitory cytokines by MDSCs, blocking of immune inhibitory pathways, delayed tumor growth in a T-cell dependent manner, and enhanced efficacy of adoptive T-cell therapy [93].

GBM cells are also able to evade the anti-tumor immune response due to altered lipid metabolism impacting the function of antigen presenting cells. Exogenous induction of lipid peroxidation and ferroptosis resulted in release of damage-associated molecular patterns from glioma cells that stimulate dendritic cell activation and maturation and can lead to activation of cytotoxic T lymphocytes by dendritic cells [94,95]. Recurrent GBM reprogram metabolic processes to enrich fatty acid oxidation which allow for adaptive tumor resistance and anti-phagocytosis [96]. Fatty acid oxidation by GBM cells activates CD47 to mediate immune escape, representing a potential target for immunotherapy [96]. Notably, one class of fatty acids, arachidonic acids, also contributes to tumor progression in GBM [97]. However, targeting these molecules with steroids has been shown to also correlate with tumor progression and inhibits responses to oncolytic adenoviral therapy and checkpoint immunotherapy [98,99]. Further understanding of the specific role of individual classes of lipids in altering the immune response in the tumor microenvironment will be needed to develop specific anti-tumoral immune strategies targeting lipid utilization in GBM.

## 6. Implications of GBM Metabolic Alterations for Specific Immunotherapy Strategies

While initially the central nervous system was thought to be an immune-privileged site, current thought points to the presence of immune surveillance in the brain following findings revealing the presence of dedicated lymphatic channels running parallel to dural venous sinuses and allowing for lymphocyte priming from antigen presenting cells in the brain [96,100]. Despite this evidence supporting the possibility of immune responses in the central nervous system, immunotherapies have not met with anywhere near the level of success in GBM that they have encountered in other cancers. Indeed, GBMs have enriched immunosuppressive myeloid, microglia, and macrophage populations and depleted tumor infiltrating lymphocytes, and thus have been characterized as “cold” tumors due to their lack of response to immunotherapy [101]. Furthermore, each component of standard of care treatment (surgery, radiation, chemotherapy, steroids) for GBM elicits immunosuppressive effects as well [102].

**Figure 3:**
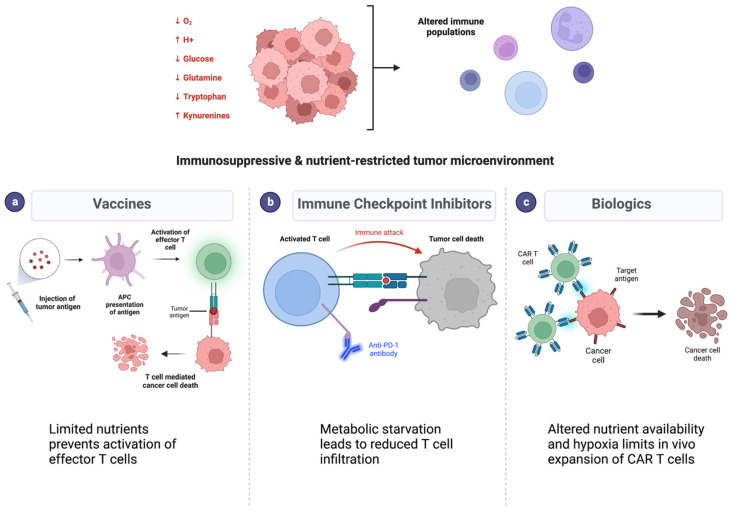
Immunosuppression and nutrient depletion in the tumor microenvironment limit efficacy of immunotherapies in glioblastoma. (**Top**) Cancer cell metabolism alters the pH, oxygen, and metabolite contents in the tumor microenvironment. (**Bottom**) Metabolic alterations result in resistance to different classes of immunotherapies such as vaccines (**a**), immune checkpoint inhibitors (**b**), and biologic therapies (**c**). Each of these mechanisms relies on an effective immune response which is diminished with metabolic alterations.

The most studied immunotherapeutic approaches for GBM are vaccines, immune checkpoint inhibitors, and biologic therapies (Figure 3). The most advanced vaccine trial in GBM, a phase 3 trial of the peptide vaccine rindopepimut, which targets EGFR variant III (EGFRvIII), relied on adaptive immunity with the tumor based on a single immunogenic peptide and failed to demonstrate improvement in survival over standard of care in EGFRvIII positive patients [103]. Peptide vaccines such as rindopepimut have poor immunogenicity on their own and require an effective T cell response to have antitumoral effects, which may limit their efficacy in GBM due to metabolic alterations in the GBM microenvironment altering effector T cell proliferation and function (Figure 3). Another vaccine-based therapy in trials was based on utilizing dendritic cells with promising early phase 3 survival data. However, dendritic cells injected into the tumor may have altered function based on altered tumoral metabolism, such as changes in lipid metabolism [104]. Promisingly, there is much more limited evidence on metabolic alterations impacting dendritic cells as compared to T cells. However, these vaccines ultimately also depend on an effective T cell response.

Immune checkpoint inhibitors targeting PD-1/PD-L1 and/or CTLA-4 are also being investigated in phase 3 trials in GBM, with initial results suggesting no clinical benefit [104,105]. While numerous factors may be responsible for the limited response observed thus far to immune checkpoint inhibitors, including the expression levels of the targets themselves, the reduced T cell infiltration in glioblastoma is a notable barrier (Figure 3). Both CTLA-4 and PD-1 mechanistically involve the T cell response—blocking CTLA-4 enhances T cell priming and blocking PD-1 enhances T cell differentiation. Addressing the metabolic restraints experienced by effector T cells within the tumor may have potential to enhance the respose to checkpoint inhibitors. Though combining metabolic targeting with immune checkpoint blockade has been investigated in other cancer types, this approach has not yet been explored in GBM. While enthusiasm for IDO inhibition combined with checkpoint inhibitors has diminished following failure in a metastatic melanoma phase 3 trial, alternate strategies could include targeting tryptophan metabolism, considering pathways that tumor cells may use to bypass IDO inhibition. Glycolytic targeting in combination with checkpoint inhibitors is another strategy that has been shown in preclinical studies as increasing T cell activation, viability, and effector function to improve the efficacy of checkpoint therapy [106].

Biologic therapies for GBM can be viral or cellular. Oncolytic viruses have overall met with limited success in GBM, and initial anti-tumor T cell immune responses generated by viral infiltration into tumor do not persist without serial treatment [107]. Metabolic alterations, particularly the limited glucose and acidosis in the tumor microenvironment, can inhibit viral replication as well as prevent the activation of CD8 T cells which are required for oncolytic viral stimulation of host anti-tumor immune responses [108]. Cellular therapies for GBM include chimeric antigen receptor (CAR) T cell therapy, in which T cells are engineered to express an activated phenotype and can be designed to recognize antigens not presented in the context of MHC molecules, potentially allowing this therapeutic approach to bypass some of the immunosuppressive metabolic limitations in the tumor microenvironment. However, in the first trial of CAR T cells directed at EGFRvIII in glioblastoma patients, tumor infiltration of CAR T cells was detected but overall survival was not affected [109]. Significantly, tumor samples from patients who underwent surgical resection after CAR T cell infusion revealed upregulation of IDO1 and increased T regulatory cells, implying the possibility of GBM escape mechanisms reinstating an immunosuppressive milieu. One potential strategy to enhance CAR T cell therapy involves culturing CAR T cells in metabolic conditions similar to the tumor microenvironment to allow for acclimation to low nutrient availability and potentially mititgate the metabolic immunosuppression in the tumor microenvironment (Figure 2). T cell expansion in media containing low levels of glutamine was shown to result in greater effective antitumor function compared to cells cultured in traditional media [110]. Ex vivo culturing of cells concurrent with glycolytic blockade demonstrated improved tumor clearance [111]. Targeting metabolic adaptations, such as increased fatty acid oxidation, in conjunction with adoptive T-cell therapy has also demonstrated success in preclinical models [93].

Metabolic pathways implicated in maintaining immunosuppression in the GBM microenvironment have been well elucidated. However, the potential for targeting these metabolic pathways to condition the tumor microenvironment to become more responsive to immunotherapies remains underexplored in GBM. Preclinical data targeting metabolic pathways in conjunction with immunotherapies largely come from models of more immunogenic cancers. This presents an attractive avenue for further study in GBM, in which modifying a largely immunosuppressive environment may meaningfully alter immunotherapeutic response. 

While this review discusses the most studied metabolic pathways with respect to immunosuppression in GBM, several other metabolic pathway alterations occur to meet the energetic demands of GBM progression. Arginine metabolism reprogramming in GBM leads to increased intake and decreased degradation of arginine by tumor cells and has been linked to impaired T cell responses due to altered bioavailablity of arginine [112]. Targeting arginine metabolism has been explored preclinically in GBM and found to synergize with radiotherapy while promoting a protumor immune population, suggesting potential to explore targeting this pathway in combination with immunotherapies in GBM [112]. Another metabolite, 2-hydroxuglutarate (2-HG), has also been implicated in both tumor growth and modulation of anti-tumor immunity through inhibition of T cell activity, though 2-HG alterations in GBM are heterogenous and epigenetically regulated [113]. In IDH-1 mutant tumors which produce 2-HG, antitumor immunity induced by an IDH-1 specific vaccine or checkpoint inhibition is improved by simultaneously downregulating 2-HG production through inhibition of the enzymatic function of mutant IDH [113]. Several other metabolites have been described as altered in GBM such as aspartate, α-ketoglutarate, and methionine; however, less evidence exists regarding the contribution of these metabolic alterations to immunosuppression. Other molecular targets in GBM have also been noted to contrastingly impact tumor cell metabolism and immunosuppression in which tumor cell metabolism is slowed while T cell activatation promotes pro-tumoral effects on the immune microenvironment [114]. These suggest a range of alternative pathways that may be implicated in the limited response to immunotherapies and warrant further preclinical study.

## 7. Conclusions

While many immunotherapies are being investigated in GBM patients, none have resulted in major improvements in survival outcomes. Negative results from phase II and phase III clinical trials of vaccines and immune checkpoint inhibitors have challenged the potential of these approaches in GBM. Future directions for immune-based strategies for glioblastoma require treatment modalities that can convert a ‘cold’ tumor with significant local immunosuppression into a ‘hot’ tumor. The uniquely immunosuppressive environment generated by tumor cellular metabolism in GBM presents an opportunity for augmenting responses to immunotherapy. Combining immunotherapy with agents that target the metabolic alterations resulting in an immunosuppressive microenvironment may have greater success in generating an antitumor immune response. These combinations should be evaluated rigorously preclinically in order to ensure that the most biologically sound combination approaches addressing the antitumor immune response along with tumor metabolism are advanced to clinical trials.
